# Oxamflatin Significantly Improves Nuclear Reprogramming, Blastocyst Quality, and *In Vitro* Development of Bovine SCNT Embryos

**DOI:** 10.1371/journal.pone.0023805

**Published:** 2011-08-30

**Authors:** Jianmin Su, Yongsheng Wang, Yanyan Li, Ruizhe Li, Qian Li, Yongyan Wu, Fusheng Quan, Jun Liu, Zekun Guo, Yong Zhang

**Affiliations:** 1 College of Veterinary Medicine, Northwest A&F University, Key Laboratory of Animal Reproductive Physiology and Embryo Technology, Ministry of Agriculture, Yangling, Shaanxi, People's Republic of China; 2 Department of Biochemistry and Molecular Biology, College of Life Sciences, Northwest A&F University, Yangling, Shaanxi, People's Republic of China; Cornell University College of Veterinary Medicine, United States of America

## Abstract

Aberrant epigenetic nuclear reprogramming results in low somatic cloning efficiency. Altering epigenetic status by applying histone deacetylase inhibitors (HDACi) enhances developmental potential of somatic cell nuclear transfer (SCNT) embryos. The present study was carried out to examine the effects of Oxamflatin, a novel HDACi, on the nuclear reprogramming and development of bovine SCNT embryos *in vitro*. We found that Oxamflatin modified the acetylation status on H3K9 and H3K18, increased total and inner cell mass (ICM) cell numbers and the ratio of ICM∶trophectoderm (TE) cells, reduced the rate of apoptosis in SCNT blastocysts, and significantly enhanced the development of bovine SCNT embryos *in vitro*. Furthermore, Oxamflatin treatment suppressed expression of the pro-apoptotic gene *Bax* and stimulated expression of the anti-apoptotic gene *Bcl-XL* and the pluripotency-related genes *OCT4* and *SOX2* in SCNT blastocysts. Additionally, the treatment also reduced the DNA methylation level of *satellite I* in SCNT blastocysts. In conclusion, Oxamflatin modifies epigenetic status and gene expression, increases blastocyst quality, and subsequently enhances the nuclear reprogramming and developmental potential of SCNT embryos.

## Introduction

Somatic cell nuclear transfer (SCNT), has successfully been used to produce cloned animals in several mammalian species [1], [2], [3], [4], [5], [6], [7], [8], [9], [10], [11], [12]. SCNT is a promising technology with potential applications in both animal science and biomedical application. However, low cloning efficiency and a high incidence of abnormalities in SCNT clones, including respiratory problems, placental deficiency, increased or decreased growth and oversized organs (i.e., large offspring syndrome), obesity, short life span, prolonged gestation, dystocia, fetal edema, hydramnios, and perinatal death [13], [14], [15], are significant barriers to the use of this technology. It is generally believed that the low cloning efficiency is mostly attributed to aberrant nuclear reprogramming of the donor cell. The nuclear reprogramming process mainly involves various epigenetic modifications, such as DNA methylation and histone modifications, which suggests that epigenetic modifications may be a key factor in improving the cloning efficiency. Hence, the prevention of epigenetic errors may improve the cloning success rate in animals.

Recently, several epigenetic remodeling drugs, such as the histone deacetylase inhibitors (HDACi) trichostatin A (TSA) [16], [17], [18], [19], [20], [21], [22], [23], [24], [25], [26], [27], [28], valproic acid (VPA) [20], [29], Scriptaid [30], [31], [32], sodium butyrate [33], [34], [35], suberoylanilide hydroxamic acid (SAHA) [28], and m-carboxycinnamic acid bishydroxamide (CBHA) [36] have been used to try and improve the developmental competence of SCNT embryos, and results have indicated that the HDACi significantly improves the *in vitro* and full-term development of SCNT embryos. Oxamflatin, another HDACi, is a novel antitumor compound, which acts by inhibiting mammalian histone deacetylase [37]. A recent study found that Oxamflatin significantly improved the cloning success rate in mice without leading to obvious abnormalities [28]. However, it is not yet known if this novel compound can also improve the development of SCNT embryos in other species, and its mechanisms of action are yet to be investigated.

Thus, we explored the effects of Oxamflatin on the *in vitro* development of bovine SCNT embryos. To investigate its effects on nuclear reprogramming of somatic cells and the way in which it improves cloning efficiency, global acetylation levels of histone H3 at lysine 9 (AcH3K9) and 18 (AcH3K18) and the quality of bovine SCNT embryos (total, trophectoderm (TE) and inner cell mass (ICM) cell numbers in blastocysts, the ratio of ICM∶TE, and the rate of apoptosis in blastocysts) were assessed by immunostaining and TUNEL assay in *in vitro*-fertilized embryos (IVF group), untreated SCNT embryos (C-NT group), and Oxamflatin-treated SCNT embryos (T-NT group). Furthermore, we analyzed the effects of Oxamflatin on the expression levels of apoptosis and development-related genes in blastocysts of the three groups using quantitative real-time PCR. The DNA methylation status of *satellite I* was also analyzed in blastocysts of the three groups.

## Results

### Experiment 1: Oxamflatin treatment improved the development of bovine SCNT embryos in vitro

**Figure 1:**
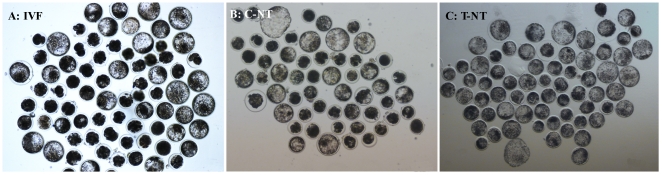
Representative photographs of bovine blastocysts. Day 7 blastocysts developed from IVF embryos (A: IVF group), 0 µM Oxamflatin treated SCNT embryos (B: C-NT group), and 1 µM Oxamflatin treated embryos (C: T-NT group). Original magnification was ×40.

To assess whether modification of acetylation could benefit early development of SCNT bovine embryos, we treated SCNT embryos with different concentrations of Oxamflatin and calculated the *in vitro* developmental rates from the 2-cell embryo to the blastocyst stage (Fig. 1, Table 1). We found that IVF and all SCNT embryos cleaved with a similar rate, around 77–81%, except 5 µM Oxamflatin-treated SCNT embryos. The effect of the Oxamflatin treatment was observed from the morula stage onwards. 0.5 µM and 1 µM Oxamflatin improved the morula and blastocyst rate. A high concentration of Oxamflatin (5 µM) was found to be toxic for development as early as the 2-cell stage.

**Table 1:** Effect of different concentration of Oxamflatin on the development of cloned bovine embryos *in vitro*.

Treatment	No. reconstructed	No. (%) ≥2-cell embryos	No. (%) ≥4-cell embryos	No. (%) ≥ morulas	No. (%) blastocysts
IVF	206	166 (79.68±2.14)^a^	144 (69.91±2.53)^a^	101 (49.03±0.53)^b^	79 (38.39±0.64)^b^
0 *µ*M	214	165 (77.23±2.46)^a^	154 (71.85±1.22)^a^	86 (40.11±0.72)^a^	65 (30.34±0.83)^a^
0.05 *µ*M	186	146 (79.09±1.83)^a^	128 (68.80±0.87)^a^	72 (38.74±1.26)^a^	55 (29.55±1.31)^a^
0.5 *µ*M	194	157 (80.02±1.11)^a^	139 (71.62±1.91)^a^	93 (47.96±2.03)^b^	77 (39.65±2.12)^b^
1 *µ*M	248	201 (81.01±1.87)^a^	181 (72.97±2.10)^a^	121 (48.83±1.38)^b^	101 (40.81±1.18)^b^
5 *µ*M	188	69 (36.73±3.13)^b^	54 (28.78±2.78)^b^	18 (9.56±3.26)^c^	11 (5.84±2.11)^c^

Four replicate experiments were performed per treatment. Numbers in parentheses represent development rates (mean ± SEM%), while other numbers represent total embryo numbers of four replicates. Development rates of 2-cell embryos, 4-cell embryos, morulas, and blastocysts were monitored at 48, 72, 120, and 168 h of culture, respectively (0 h being the time embryos were transferred to G1.3).

Within a column, developmental rates with different superscripts are significantly different from each other (P<0.05).

To optimize the treatment of Oxamflatin, we also tested the developmental rates of blastocyst with various incubation times. The optimum effect was reached when the SCNT embryos were treated with 1 µM Oxamflatin post-ionomycin for 12 h. The developmental rates of blastocyst were 29.05±2.31%, 30.64±0.78%, 40.81±1.18%, 34.34±1.24%, and 31.04±2.61% for incubation times of 0, 6, 12, 18, and 24 h, respectively.

### Experiment 2: Oxamflatin treatment increased global histone acetylation levels of SCNT embryos

**Figure 2:**
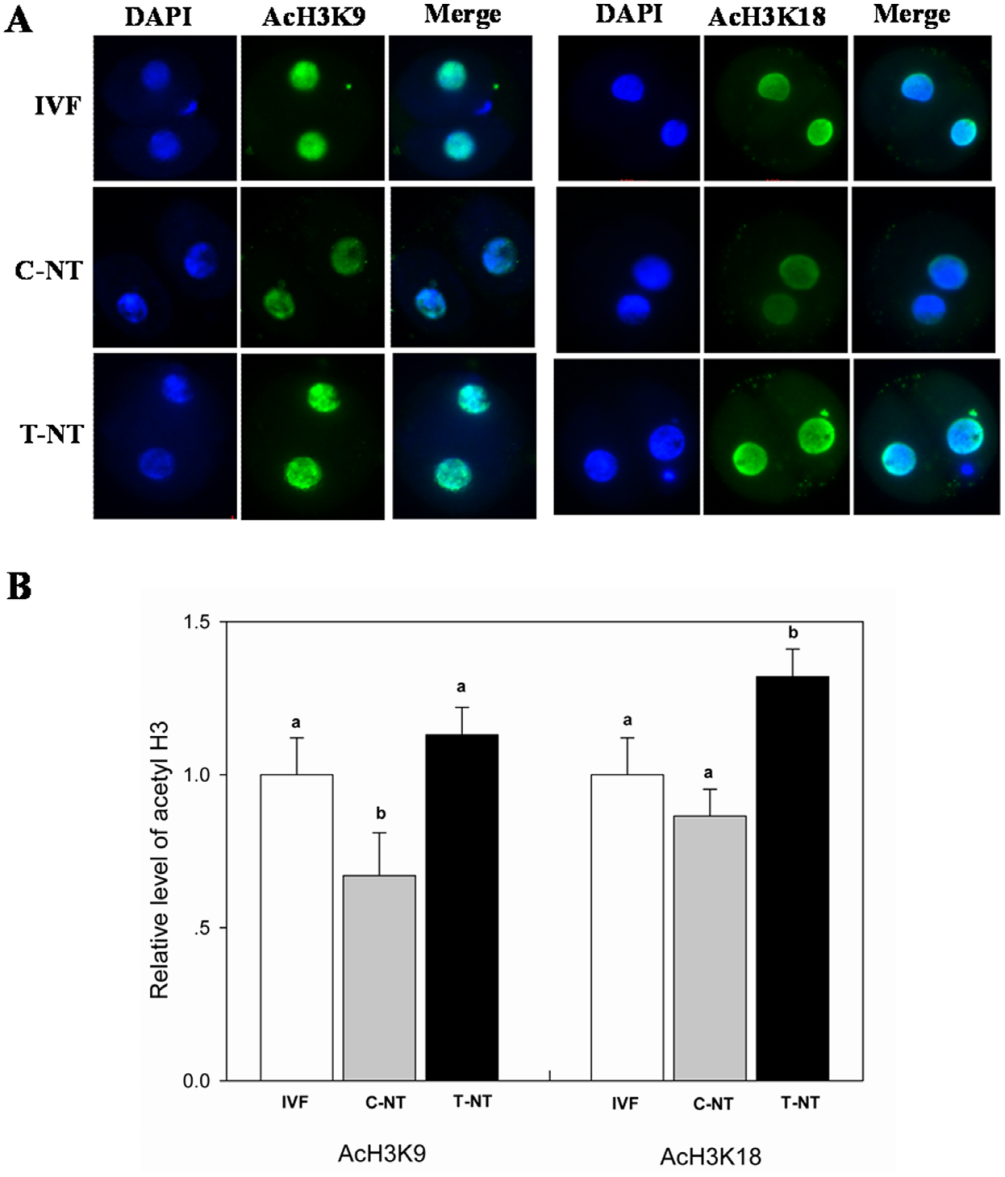
The global AcH3K9 and AcH3K18 levels in 2-cell stage embryos. (A) Staining of AcH3K9 and AcH3K18 (green) in IVF, 0 µM Oxamflatin treated SCNT (C-NT), and 1 µM Oxamflatin treated embryos (T-NT) at the 2-cell stage. Each sample was counterstained with DAPI to visualize DNA (blue). Original magnification was ×200. (B) Quantification of AcH3K9/DNA and AcH3K18/DNA signal intensities in IVF (open bars), C-NT (gray bars), and T-NT (black bars) embryos. Labeling intensity was expressed relative to that of the IVF embryos (set as 100%). Values with different superscripts differ significantly (P<0.05). The experiments were replicated 3 times. In each replication, n = 10–15 per group.

**Figure 3:**
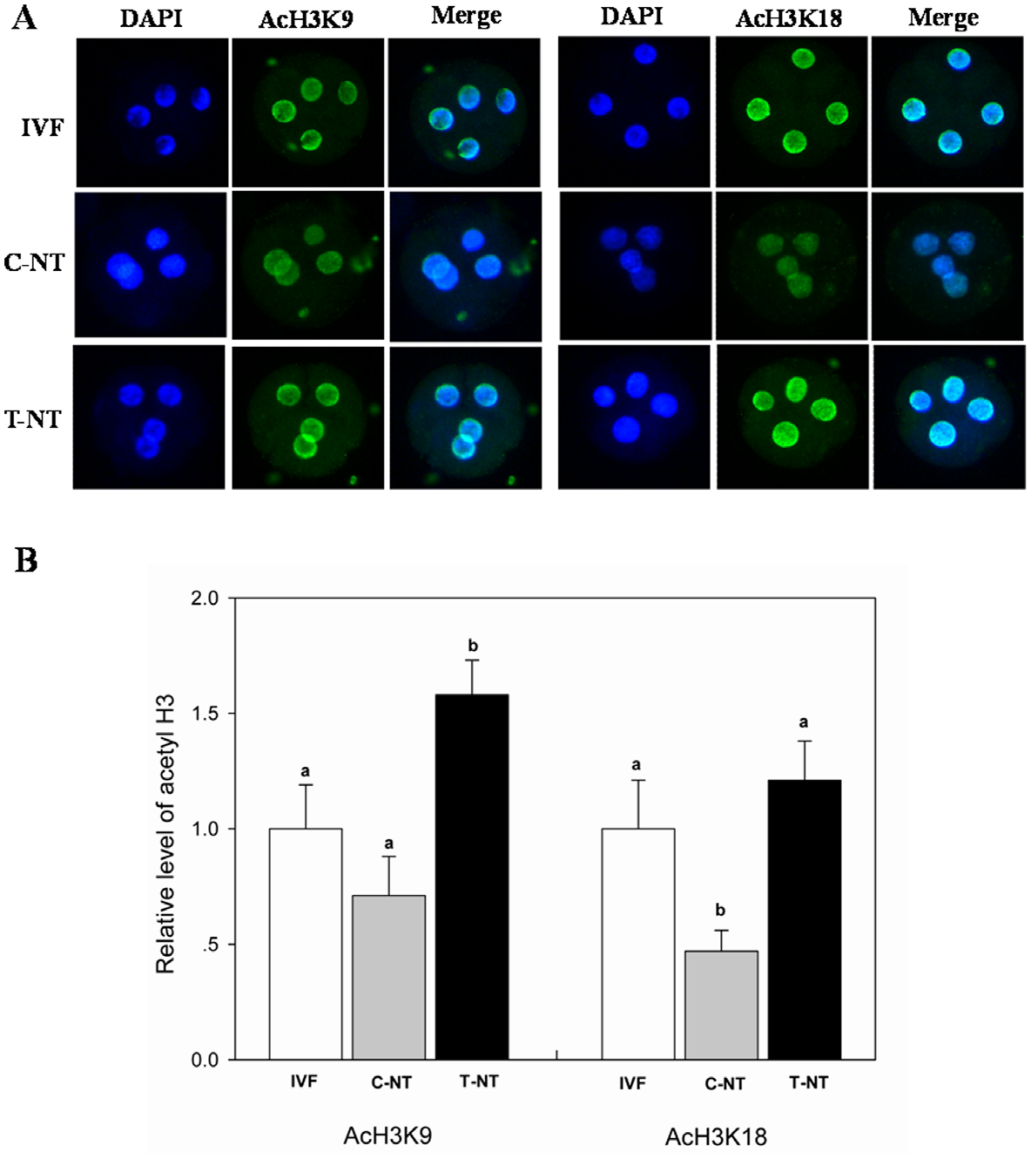
The global AcH3K9 and AcH3K18 levels in 4-cell stage embryos. Details are described in the legend to Fig. 2.

**Figure 4:**
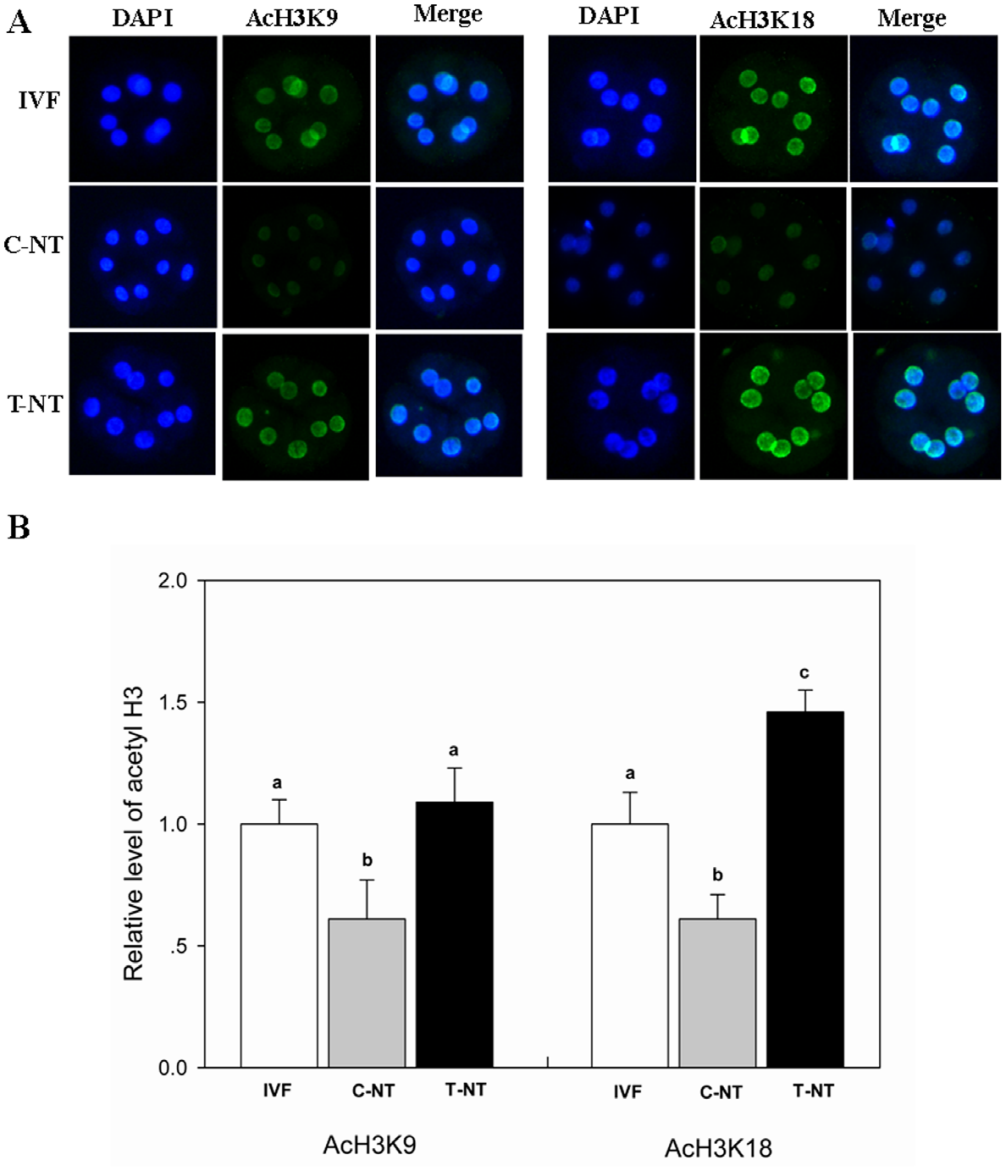
The global AcH3K9 and AcH3K18 levels in 8-cell stage embryos. Details are described in the legend to Fig. 2.

**Figure 5:**
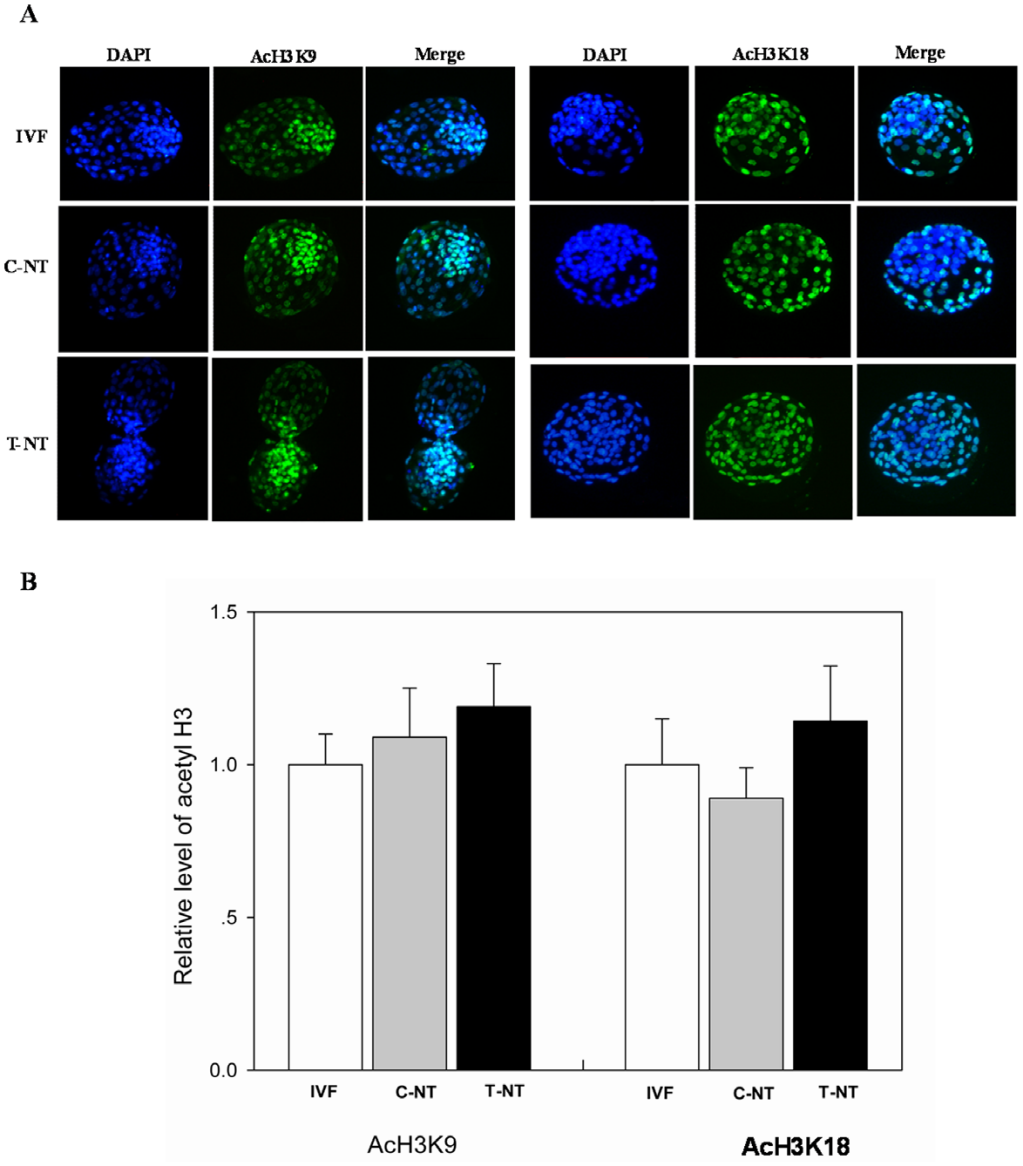
The global AcH3K9 and AcH3K18 levels in blastocysts. Details are described in the legend to Fig. 2.

To find the way in which Oxamflatin treatment improved the developmental potential of SCNT embryos, the acetylation levels of two epigenetic markers, H3K9 and H3K18, were studied in 2-cell, 4-cell, 8-cell, and blastocyst stage embryos. No signals were detected in the embryos stained without first or secondary antibodies, indicating the specificity of staining of the first antibody (data not shown). As shown in Fig. 2, 3, and 4, Oxamflatin treatment increased AcH3K9 and AcH3K18 levels in 2-cell, 4-cell, and 8-cell stage embryos. However, at the blastocyst stage, no differences in AcH3K9 and AcH3K18 levels were observed among the groups (Fig. 5).

### Experiment 3: Oxamflatin treatment increased total cell numbers, the number of ICM cells, and the ICM∶TE ratio in SCNT blastocysts

**Figure 6:**
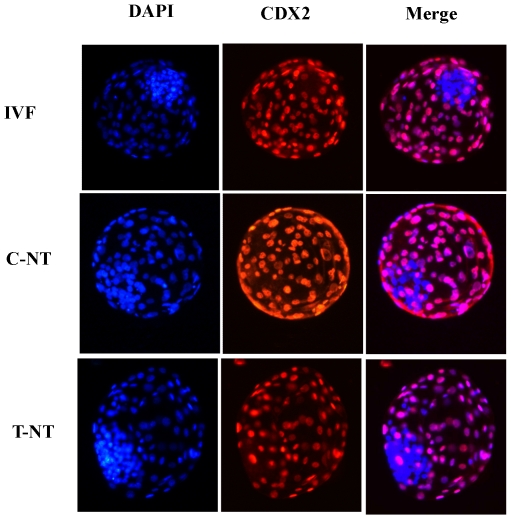
Immunostaining of CDX2. Each blastocyst in the IVF, 0 µM Oxamflatin treated SCNT (C-NT), and 1 µM Oxamflatin treated SCNT groups (T-NT) was stained with DAPI and CDX2, a marker for trophectoderm. Original magnification was ×200. n = 30, 38, and 44 in the IVF, C-NT, and T-NT group, respectively.

To investigate the mechanism behind the improved development of SCNT embryos after Oxamflatin treatment, we measured the total cell numbers (DAPI staining), the expression pattern of markers for TE (CDX2 staining), and the estimated ICM cell number (total cells minus TE cells) in blastocysts of the three groups. The total number of blastomeres, ICM cells, and the ratio of ICM∶TE were significantly higher in the Oxamflatin-treated blastocysts (T-NT group) than in untreated ones (C-NT group) (P<0.05). TE cell numbers were not statistically different among the three groups (Fig. 6; Table 2).

**Table 2:** Characterization of day 7 bovine blastocysts.

Groups	n	DAPI staining		CDX2 staining		DAPI/CDX2 staining		
		Total no. of cells	Range	No. of TE cells	Range	No. of ICM cells	Range	ICM: TE (%)
IVF	30	100.73±8.92^ab^	67–159	71.47±5.63	46–111	29.27±3.50^a^	16–57	39.84±2.13^a^
C-NT	38	85.26±5.32^b^	60–149	64.79±3.56	48–105	20.47±2.01^b^	13–44	31.10±1.79^b^
T-NT	44	111.45±7.46^a^	70–158	76.32±5.02	52–114	35.14±2.61^a^	18–56	45.87±1.61^c^

The cell numbers in blastocysts were estimated by counting the total number of nuclei using DAPI. The number of trophectoderm (TE) nuclei was estimated using immunostaining for CDX2. The ICM cell number was assessed as the total number of nuclei minus the number of TE nuclei. The data are shown as Mean ± SEM.

Within columns, values with different superscripts are significantly different from each other (P<0.05).

### Experiment 4: The number of apoptotic cells was lower in Oxamflatin-treated blastocysts

**Figure 7:**
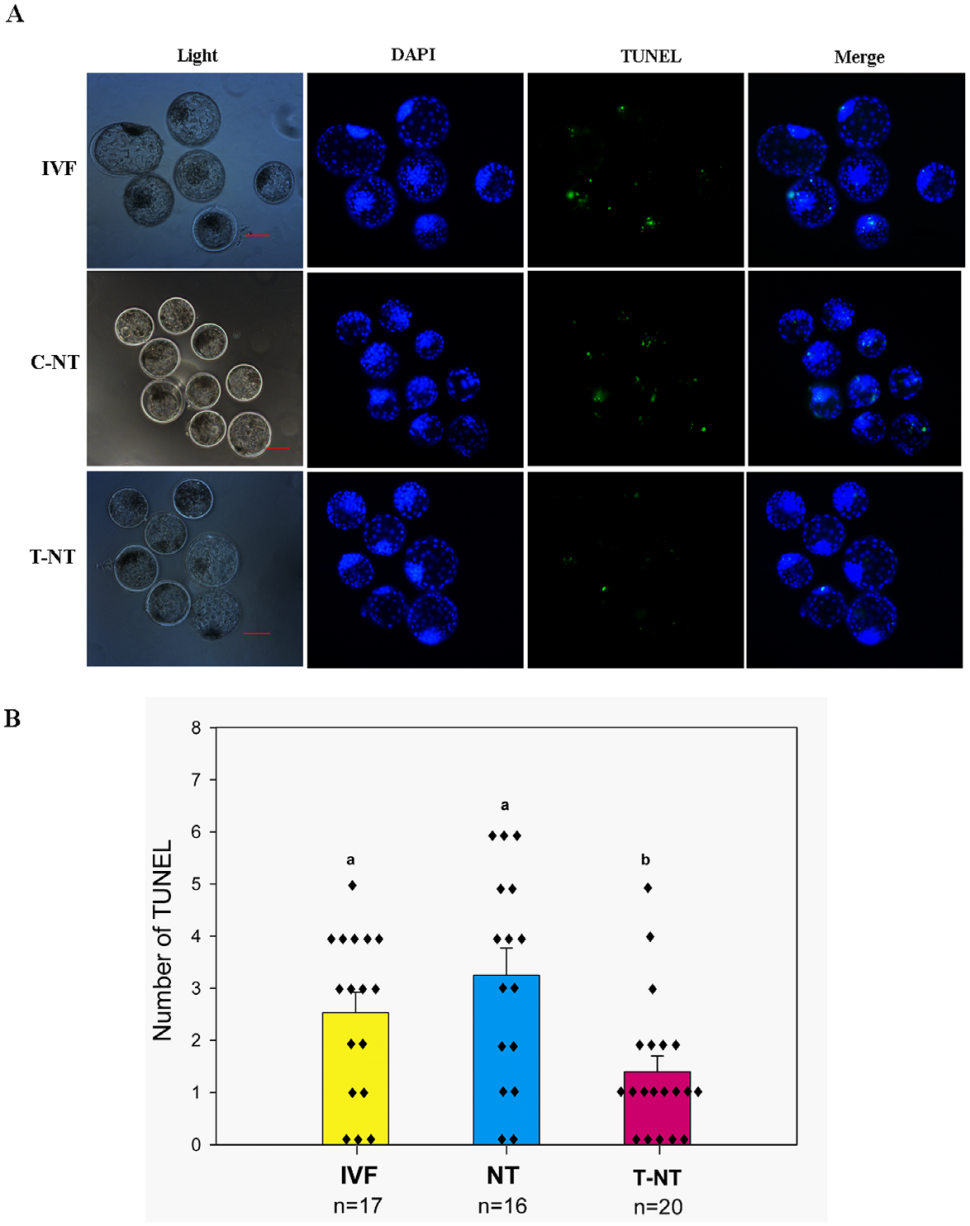
Incidence of apoptosis in blastocysts. (A) TUNEL assay of blastocysts (green). Each sample was counterstained with DAPI to visualize DNA (blue). Original magnification was ×40. (B) Number of apoptotic cells in each blastocyst. Values with different superscripts differ significantly (P<0.05). n = 16–20.

To determine if the improvement in SCNT embryo development was reflected in blastocyst quality, the number of apoptotic cells was estimated by TUNEL assay. As shown in Fig. 7, the number of apoptotic cells in SCNT blastocysts was significantly lower in the T-NT group than in the C-NT and IVF groups (P<0.05).

### Experiment 5: Oxamflatin treatment affected relative expression of apoptosis and development-related genes

**Figure 8:**
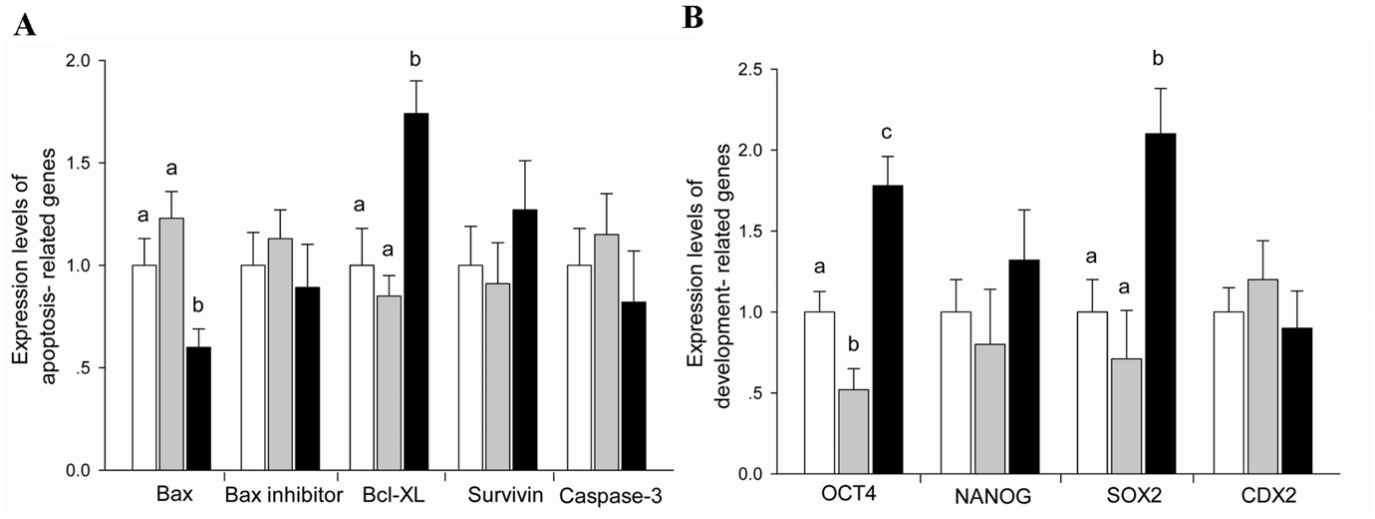
Relative abundance of apoptosis and development-related genes. Relative expression levels of apoptosis (**A**) and development (**B**) related genes in single day 7 IVF (open bars), C-NT (gray bars), and T-NT (black bars) blastocysts. Values with different superscripts differ significantly (P<0.05); n = 5–8.

Relative expression levels of 9 different genes were analyzed in IVF, C-NT, and T-NT blastocysts using quantitative real-time PCR (Fig. 8). The expression level of *Bax* was lower in T-NT blastocysts than in C-NT blastocysts (P<0.05). The expression levels of *Bcl-XL*, *OCT4* and *SOX2* were significantly higher in T-NT blastocysts than in C-NT blastocysts (P<0.05). The expression level of *OCT4* was lower in the C-NT group than in the IVF group (P<0.05). There were no significant differences in the expression of *Bax inhibitor*, *Survivin*, *Caspase-3*, *NANOG*, and *CDX2* among the three groups.

### Experiment 6: Oxamflatin treatment reduced DNA methylation levels in the satellite I region

**Figure 9:**
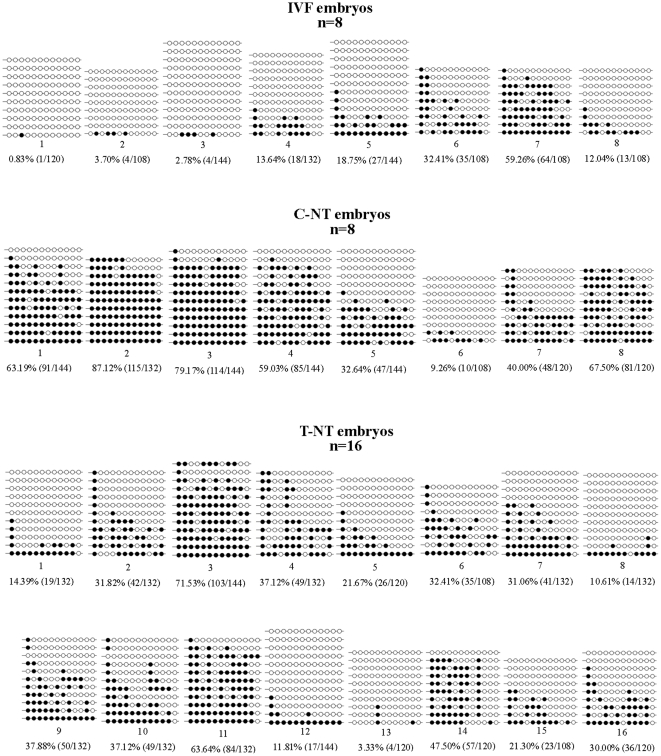
Methylation profiles of 12 CpGs in the *satellite I* region, analyzed by bisulfite sequencing. White and black circles represent unmethylated and methylated CpGs, respectively. Horizontal lines of circles represent one separate clone that was sequenced (9–15 for each sample). Lollipop diagrams were generated with the BIQ Analyzer software [69]. For each sample, the methylation data were analyzed by computing the percentage of methylated CpGs of the total number of CpGs. n = 8, 8, and 16 in the IVF, C-NT, and T-NT group, respectively.

The DNA methylation status of *satellite I* was analyzed in blastocysts by bisulfite sequencing (Fig. 9). The *satellite I* sequence of IVF blastocysts (17.92±6.94%) and T-NT blastocysts (31.45±4.61%), had significantly lower methylation levels than that of C-NT blastocysts (53.99±9.11%, P<0.05).

## Discussion

Somatic cells can be reprogrammed into pluripotent cells with various methods, such as induction of ectopic expression of transcription factors (induced pluripotent stem cells; iPSCs) or nuclear transfer into enucleated oocytes using SCNT [38]. It was recently shown that iPSCs display more genetic and epigenetic abnormalities than ESCs or fibroblasts – the cells from which they originate [39], [40], [41]. Thus, pluripotent cells generated by SCNT technology may have greater therapeutic potential. Besides therapeutic cloning, SCNT is also a promising technology with potential applications in species preservation, livestock propagation, transgenic research, human xenotransplantation, and disease models. The cloning efficiency, however, remains low.

Recently, investigations have focused on the ability of histone deacetylase inhibitors to improve SCNT efficiency. However, studies exploring the mechanism with which HDACi enhances the epigenetic remodeling ability of somatic cell nuclei in SCNT embryos are scarce.

In this study, we examined if Oxamflatin treatment can improve somatic nucleus reprogramming and development of bovine SCNT embryos *in vitro*. Furthermore, we also explored, for the first time, Oxamflatin's possible mechanisms of action. We found that Oxamflatin treatment after SCNT modified the epigenetic status and gene expression in SCNT embryos, increased blastocyst quality, and significantly improved the subsequent development of bovine SCNT embryos.

The best protocol for Oxamflatin treatment in cattle was found to be: (1) Oxamflatin concentration of 0.5–1 µM, as Oxamflatin becomes effective from 0.5 µM but shows toxicity at 5 µM, (2) continuous exposure of reconstructed oocytes to Oxamflatin post-ionomycin for 12 h (4 h in DMAP containing Oxamflatin and 8 h in G1.3 medium containing Oxamflatin).

A recent study in mice showed that Oxamflatin treatment (1 µM Oxamflatin for 9 h after nuclear transfer) significantly improved the *in vitro* and full-term development of cloned mice [28]. In the present study, we found that Oxamflatin treatment (1 µM Oxamflatin post-ionomycin for 12 h) also significantly improved the *in vitro* development of cloned cattle. Our previous studies on bovine cloning showed that treatment with 5-aza-dC and TSA dramatically improved the development of SCNT bovine embryos *in vitro* [19] and *in vivo* [42], thereby significantly increasing bovine cloning efficiency from 2.6% to 13.4% (number of surviving calves at 60 days of birth/number of recipient cows) [42]. Accordingly, we infer that epigenetic modification drugs may also have effects on the *in vivo* and full-term development of cloned embryos.

Histone acetylation, one of two main types of epigenetic marker, plays a significant role in the process of reprogramming and affects the development of SCNT embryos [19], [23], [33], [36], [43], [44]. Therefore, we studied the acetylation level of histone H3 in SCNT embryos and found that Oxamflatin treatment increased both AcH3K9 and AcH3K18 levels in 2-cell, 4-cell, and 8-cell SCNT embryos. It is well accepted that increasing global acetylation of histones by HDACi alleviates transcriptional repression by facilitating chromatin remodeling and relieving methylated CpG sites [45], [46]. It is believed that hyperacetylation of histones facilitates the access of various factors to nucleosomes [32], [47], [48]. Therefore, one of the ways in which Oxamflatin treatment improves the developmental potential of SCNT embryos may be that the increased histone acetylation level, caused by inhibition of HDAC activity, may facilitate chromatin remodeling and access of reprogramming-related factors to nucleosomes, alleviating transcriptional repression.

DNA methylation, another key epigenetic factor, modifies and regulates the chromatin structure and also plays a crucial role in somatic nuclear reprogramming. It was found that TSA not only modifies histone acetylation but also potentially induces DNA demethylation [49]. Bovine SCNT embryos were found to have aberrant hypermethylation in the *satellite I* region [50]. Wee et al. [43] and we [51] have found that HDACi induce DNA demethylation in the *satellite I* region. Therefore, we further investigated if Oxamflatin treatment affects DNA methylation in the *satellite I* region of SCNT embryos. Interestingly, most Oxamflatin treated SCNT embryos had significantly lower DNA methylation levels in the *satellite I* region than non-treated SCNT embryos. Like TSA, Oxamflatin treatment seems to have a “correcting” effect on the DNA methylation status of the *satellite I* region.

The cell number, especially the ratio of ICM∶TE cells, is one of the criteria for assessment of blastocyst quality [52], [53]. Aberrant allocation of ICM and TE cells in SCNT embryos at preimplantation stages may cause placental abnormalities and early fetal loss [54]. Therefore, we counted total blastomere cell numbers, TE and the estimated ICM cell numbers and measured the ratio of ICM∶TE in blastocysts. Interestingly, Oxamflatin treatment increased the total number of cells and the number of ICM cells in SCNT blastocysts. The ratio of ICM∶TE was also increased in the Oxamflatin treated group. To elucidate the mechanism behind this, we measured the relative expression levels of 4 the development-related genes *OCT4*, *NANOG*, *SOX2*, and *CDX2*, and found up-regulated expression of *OCT4* and *SOX2* in the Oxamflatin treated group relative to the non-treated group.

*OCT4* is a key regulator of pluripotency that is important for maintaining ICM cell fate and pluripotency of ES cells. It has been reported that *OCT4* is not only expressed in ICM, but also in TE cells of bovine embryos [55] . Lower expression of *OCT4* was found in bovine SCNT blastocysts than in their IVF counterparts [51], [56], [57]. In this study, we found similar results. However, Oxamflatin treated SCNT blastocysts had higher *OCT4* expression levels than both non-treated SCNT and IVF blastocysts.

*SOX2* is another vital regulator of pluripotency. *SOX2* acts synergistically with *OCT4* in activating *OCT*–*SOX* enhancers, which regulate the expression of *Nanog*, *OCT4* and *SOX2* itself [58]. In this study, we found that the Oxamflatin treatment up-regulated the expression of *SOX2* in the bovine SCNT blastocysts.

Both pluripotency-related genes *OCT4* and *SOX2* are important for maintaining ICM cell fate. Therefore, the up-regulated expression of these two genes in the treated SCNT blastocysts may be associated with the higher ICM∶TE ratio. We previously found that 5-aza-dC and TSA treatment also increases the expression levels of *OCT4* and *SOX2* [51] and the number of ICM cells in the bovine SCNT blastocysts [19].

Apoptosis is another criterion for evaluation of blastocyst quality, as it eliminates cells with nuclear or chromosomal abnormalities [59]. In bovine embryos, apoptosis can be detected in embryos after the 8-cell stage [60]. The high rate of apoptosis in SCNT blastocysts is correlated with a decrease in the total cell number [53]. In this study, the number of apoptotic cells was significantly lower in the Oxamflatin treated blastocysts than in the non-treated and IVF blastocysts. This suggests that Oxamflatin improves the quality of SCNT embryos by reducing cell death in the embryos. This observation is similar to that reported in cloned mice [28]. TSA, another important HDACi, also suppresses apoptosis in bovine SCNT embryos [61] and rat kidney cells [62].

To find the cause of the decreased apoptosis rate, we further analyzed the relative expression levels of five apoptosis-related genes (*Bax*, *Bax inhibitor*, *Survivin*, *Bcl-XL*, and *Caspase-3*). During embryogenesis, the pro- and anti-apoptotic members of the Bcl-2 family of proteins regulate the pathways of apoptosis. The pro-apoptotic gene *Bax* is a positive regulator of apoptosis, whereas the anti-apoptotic gene *Bcl-XL* acts to protect against apoptosis. In the present study, there was a lower expression level of *Bax* and a higher expression level of *Bcl-XL* in Oxamflatin treated SCNT blastocysts than in non-treated blastocysts, which may have contributed to the reduced apoptosis of cells in Oxamflatin treated blastocysts compared with those in non-treated ones.

In summary, the present study indicates that the histone deacetylase inhibitor Oxamflatin affects the expression of apoptosis and development-related genes, modifies global histone acetylation and DNA methylation in *satellite I* region, increases the total and ICM cells numbers of SCNT blastocysts, reduces cell death in SCNT embryos, and subsequently enhances the nuclear reprogramming and developmental potential of bovine SCNT embryos.

## Materials and Methods

### Ethics statement

The entire experimental procedure was approved by the Animal Care Commission of the College of Veterinary Medicine, Northwest A&F University. Bovine ovaries of slaughtered mature cattle were collected from Tumen abattoir, a local slaughterhouse of Xi'An, P.R. China. A newborn female Holstein calf was obtained for nuclear donor cell cultures and Beef-breed Angus cows were obtained for recipient animals from Yangling Keyuan Cloning Co., Ltd.

### Chemicals

All chemicals and reagents were purchased from Sigma-Aldrich (St. Louis, USA) unless specifically stated otherwise. Disposable, sterile plasticware was purchased from Nunclon (Roskilde, Denmark).

### Nuclear donor cell preparation

Nuclear donor cell cultures were established from the ear skin of a newborn female Holstein calf as described previously [51]. Briefly, after the hair was removed, the ear notch was rinsed four times with phosphate-buffered saline (PBS), and minced into 1 mm^3^ pieces. The tissue pieces were cultivated for 1–2 weeks in 60 mm Petri dishes with DMEM (Gibco, Grand Island, USA) containing 10% FBS (Gibco), 1 mM sodium pyruvate, 100 mg/mL streptomycin and 100 IU/mL penicillin. When fibroblast cells were at 90% confluence, cells were trypsinized, rinsed, and recultivated in 3 new 60 mm Petri dishes for further passaging. Nuclear donor cells for SCNT were derived from passages 2 to 4 and cultured in serum-starved medium (0.5% FBS) for 2 days.

### Oocyte collection and *in vitro* maturation (IVM)

Oocyte collection and *in vitro* maturation (IVM) were preformed as described previously [51], [63], [64]. Briefly, bovine ovaries were transported from the slaughterhouse to the laboratory within 4 h after the animal was killed in a thermos bottle with sterile saline at 20–25°C. A 12-gauge needle attached to a 10 mL syringe was used to aspirate cumulus–oocyte complexes (COCs) from antral follicles with a diameter of between 2 and 8 mm. COCs were recovered and washed in PBS containing 5% (v/v) FBS. Only oocytes surrounded by a minimum of 3 cumulus cell layers and with uniform cytoplasm were selected, washed in PBS containing 5% (v/v) FBS, and cultured for 20 h in bicarbonate-buffered tissue culture medium 199 (TCM-199, Gibco) containing 10% (v/v) FBS, 1 µg/mL 17 β-estradiol, and 0.075 IU/mL Human Menopausal Gonadotropin (HMG) in 95% humidified air with 5% CO_2_ at 38.5°C.

### SCNT, activation, and culture of SCNT embryos

**Figure 10:**
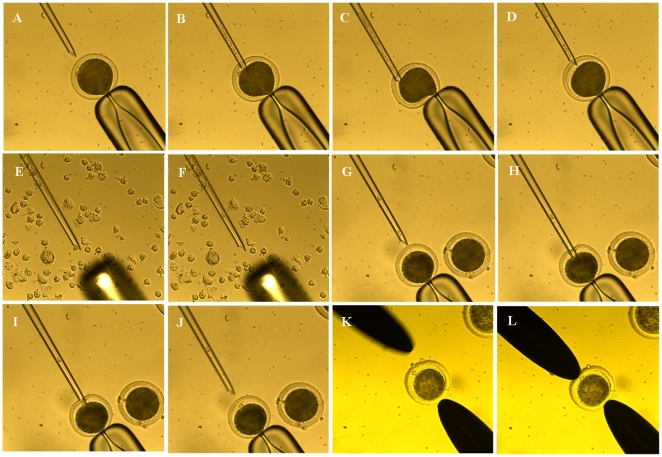
Somatic cell nuclear transfer procedure. (A–D) The first polar body and a small amount of surrounding cytoplasm was aspirated using a 20 µm inner diameter glass pipette; (E–J) A single disaggregated donor cell was injected into the pre-vitelline space of the enucleated oocytes; (K, L) The oocyte-cell fusion.

SCNT, activation of reconstructed embryos, and culture of SCNT embryos were achieved as described previously [51], [63], [64]. Briefly, after IVM for 20 h, the cumulus cells of COCs were dispersed by vortexing for 3 min in PBS containing 0.1% bovine testicular hyaluronidase in 1.5-mL centrifuge tubes. Only oocytes having an extruded first polar body and with uniform ooplasm were selected and stained with 10 µg/mL Hoechst 33342 for 10 min. Enucleation was performed using a 20 µm inner diameter glass pipette by aspirating the first polar body and a small amount of surrounding cytoplasm in PBS microdrops containing 7.5 µg/mL cytochalasin B and 10% FBS (Fig. 10A–D). The expelled cytoplasm was surveyed under ultraviolet radiation to verify that the nuclear material had been removed. A single disaggregated donor cell was injected into the pre-vitelline space of the enucleated oocytes (Fig. 10E–J). The oocyte-cell fusion was performed using a pair of platinum electrodes connected to a micromanipulator in microdrops of Zimmermann's fusion medium, and a double electrical pulse of 35 V for 10 µs was used for fusion (Fig. 10K, L). Reconstructed SCNT embryos were kept in synthetic oviductal fluid (SOFaa) containing 5 µg/mL cytochalasin B for 2 h until activation. The mSOF medium was prepared according to the formula described previously [65] and supplemented with 8 mg/mL of bovine serum albumin, 1% MEM nonessential amino acid solution and 2% BME essential amino acid solution. Activation of reconstructed embryos was performed in 5 µM Ionomycin for 4 min followed by 4 h exposure to 1.9 mM dimethynopyridine (DMAP) in SOFaa. After activation, embryos were cultured in G1.3/G2.3 sequential media (Vitrolife AB, Gothenburg, Sweden). Droplets of 150 µL G1.3 were prepared in a 35-mm cell culture dish under mineral oil and equilibrated for 2 h before loading of embryos (20 embryos/microdrop). Embryos were transferred to G2.3 droplets on day 3 of culture (day 0 being the day of SCNT).

### Oxamflatin treatment protocol

Oxamflatin was dissolved in dimethyl sulfoxide (DMSO) to achieve a stock solution of 10 mM (1 mg Oxamflatin in 291 µL DMSO), and stored at −20°C. Working solutions were freshly prepared just before use. First, the 10 mM Oxamflatin stock solution was diluted in SOFaa to obtain 100 µM Oxamflatin. Then 100 µM Oxamflatin was added to the activation or culture media according to the experimental protocol. For the control group (C-NT group), DMSO was added to the culture medium at the same concentration as used in the other treatments.

Various concentration: Immediately following ionomycin treatment, SCNT embryos were incubated for 4 h in SOF medium with 1.9 mM DMAP containing 0, 0.05, 0.5, 1 or 5 µM Oxamflatin. SCNT embryos were then incubated for another 8 h in G1.3 medium supplemented with 0.05, 0.5, 1 or 5 µM Oxamflatin. After treatment, embryos were washed twice with G1.3, and cultured in 150 µL drops of G1.3 medium in a humidified atmosphere with 5% CO_2_ in air at 38.5°C.

Various incubation times: 1 µM Oxamflatin was added post-ionomycin for 0, 6, 12, 18 or 24 h.

### In vitro fertilization

IVF was carried out in accordance with the methods of our previous study [51].

### Immunofluorescence staining of embryos

Embryos were washed 3 times (5 min each) in PBS containing 0.2% PVA, and fixed in Immunol Staining Fix Solution (Beyotime, P0098, Jiangsu, China) for 1 h. All steps were performed at room temperature unless otherwise stated. Embryos were permeabilized with 0.2% Triton X-100 in PBS for 30 min. After 3 washes, they were blocked in the Immunol Staining Blocking Solution (Beyotime, P0102) for 12 h at 4°C and then incubated with the first antibodies for 12 h at 4°C. Antibodies against acetylated histones were diluted 1∶500 (AcH3K9, ab10812, Abcam, Cambridge, UK; AcH3K18, ab1191, Abcam), and anti-CDX2 mouse monoclonal antibody (BioGenex, Inc., San Ramon, CA) was diluted 1∶200 using Immunol Staining Primary Antibody Dilution Solution (Beyotime, P0103). After 3 washes, the embryos were treated with secondary antibodies of Alexa Fluor 488-labeled Goat Anti-Rabbit IgG (Beyotime, A0423) for AcH3K9 and AcH3K18 or Alexa Fluor 555-labeled Goat Anti-Mouse IgG (Beyotime, A0459) for CDX2. The secondary antibodies were diluted 1∶500 with Immnol Staining Secondary Antibody Dilution Solution (Beyotime, P0108). Finally, the DNA was stained with 4,6-diamidino-2-phenylindole (DAPI) (Beyotime, C1005) for 3 min, and samples were mounted on glass slides with a drop of Antifade Mounting Medium (Beyotime, P0126) and analyzed using a Nikon eclipse Ti-S microscope equipped with a 198 Nikon DS-Ri1 digital camera (Nikon, Tokyo, Japan). The experiments were replicated 3 times. In each replication, 10 to 15 embryos per group were processed. The intensity of AcH3K9 or AcH3K18 (green fluorescence) was analyzed using MetaMorph software (Version 6.1; Universal Imaging Corporation) and compared with that of DAPI signal (blue fluorescence) as described previously [33], [44]. To quantify fluorescence intensity, the intensity levels of C-NT and T-NT embryos were presented relative to the mean intensity level of IVF embryos.

### Apoptosis assays

A DeadEnd Fluorometric TUNEL System (Promega, Madison, WI) was used for apoptosis assays. According to the instruction manual, day 7 blastocysts were washed 3 times (5 min each) in PBS containing 0.2% PVA and fixed in Immunol Staining Fix Solution (Beyotime) for 1 h. All steps were performed at room temperature unless stated otherwise. Embryos were permeabilized with 0.2% Triton X-100 in PBS for 20 min. After 3 washes, embryos were equilibrated with equilibration buffer for 8 min. They were then incubated with rTdT incubation buffer (45 µl equilibration buffer, 5 µl Nucleotide Mix, 1 rTdTµl Enzyme) in the dark for 1 h at 37°C. The tailing reaction was terminated in 2× standard saline citrate for 15 min. Finally, the DNA was stained with DAPI (Beyotime) for 3 min, and samples were mounted on glass slides with a drop of antifade mounting medium (Beyotime) and analyzed using the Nikon eclipse Ti-S microscope equipped with the 198 Nikon DS-Ri1 digital camera (Nikon). The experiments were replicated 3 times, and a total number of 17, 16, and 20 embryos were processed in IVF, C-NT, and T-NT groups, respectively.

### Quantitative real-time PCR

A single day 7 blastocyst was used per sample, and 5 to 8 embryos were used for each group. The total RNA of the embryos was isolated using the Cells-to-Signal™ Kit (Ambion Co., USA) according to the manufacturer's protocol. The RT reaction was achieved using the M-MLV RT included in the Cells-to-Signal Kit. The mRNA levels were quantified using SYBR Premix ExTaq™ II (TaKaRa, Japan) on a CFX96 real-time PCR detection system (Bio-Rad) at the following thermal cycling conditions: 95°C for 1 min, followed by 40 PCR cycles of 95°C for 5 s, 50–60°C (Table 3) for 30 s, and 72°C for 30 s. The melting protocol was a step cycle starting at 65°C and increasing to 95°C with 0.5°C/5 s increments. The primer sequences for all genes were synthesized according to previous reports [22], [66], [67] (Table 3). Transcripts were quantified in 3 replicates for each sample and calculated relative to the transcription of the housekeeping gene, Histone 2a (H2A) in every sample. The specificity of the PCR reaction was confirmed by gel electrophoresis on a 2.5% agarose gel and by a single peak in the melting curve. For the negative controls, dH_2_O replaced cDNA in the real-time reaction tubes.

**Table 3:** Primer sequences for BS-PCR and real-time PCR.

Genes	Primer sequences (5′-3′)	*T*_ann_ a (°C)
satellite I	F^b^: AATACCTCTAATTTCAAACT	46
	R^c^: TTTGTGAATGTAGTTAATA	
OCT4	F: CCACCCTGCAGCAAATTAGC	60
	R: CCACACTCGGACCACGTCTT	
NANOG	F: CGTGTCCTTGCAAACGTCAT	60
	R: CTGTCTCTCCTCTTCCCTCCTC	
SOX2	F:GGTTGACATCGTTGGTAATTTATAATAGC	60
	R: CACAGTAATTTCATGTTGGTTTTTCA	
CDX2	F: GCAAAGGAAAGGAAAATCAACAA	60
	R: GGGCTCTGGGACGCTTCT	
Bax	F: GCTCTGAGCAGATCAAG	56
	R: AGCCGCTCTCGAAGGAAGTC	
Bax inhibitor	F: GCTCTGGACTTGTGCATT	56
	R: GCCAAGATCATCATGAGC	
Survivin	F: CCTGGCAGCTCTACCTCAAG	56
	R: TAAGTAGGCCAACACGAAAG	
Bcl-XL	F: GGTATTGGTGAGTCGGATCG	55
	R: CAAGACGACCCGAGTGAGAA	
Caspase-3	F: CGATCTGGTACAGACGTG	50
	R: GCCATGTCATCCTCA	
H2A	F: GTCTTGGAGTACCTGACCGC	56
	R: ACAACGAGGGCTTCTTCTGA	

a Annealing temperature.

b Forward primer.

c Reverse primer.

The 2^−ΔΔCT^ method [68] was used to quantify the relative mRNA levels. For ease of comparison, the average expression level of each gene from IVF group was set as 1.

### Bisulfite sequencing analysis

A single day 7 blastocyst was used per sample, and 8, 8, and 16 embryo samples were processed for IVF, C-NT, and T-NT groups, respectively. Genomic DNA was extracted from the embryos and subjected to sodium bisulfite treatment using the EZ DNA Methylation-Direct™ Kit (Zymo Research, USA) in accordance with the instruction manual with minor modifications as described previously [51], [63]. Briefly, each single blastocyst was washed and transferred to 20 µL digestion mixture. After incubation for 3 h at 50°C, the digested sample was added to 130 µL CT Conversion Reagent for bisulfite conversion and incubated at 98°C for 8 min and 64°C for 3.5 h. Modified DNA was then desalted, purified, and finally eluted with 15 µL of elution buffer. Subsequently, Bisulfite Sequencing PCR (BS-PCR) was immediately carried out using 2 µL of modified DNA per PCR run. The primers of *satellite I* were synthesized as described previously [50] (Table 3). The BS-PCRs were performed using the Hot Start DNA polymerase Zymo Taq™ premix (Zymo Research, USA) with a DNA engine (MJ Research) using the following program: 4 min at 95°C, followed by 40 cycles of denaturation for 30 s at 95°C, annealing for 30 s at 46°C, extension for 20 s at 72°C, and a final extension at 72°C for 7 min. The PCR products were gel-purified using the TIANgel Midi Purification Kit (Tiangen, China). Purified fragments were subcloned into pMD18-T vectors (TaKaRa, Japan). The clones confirmed by PCR were selected for DNA sequencing (BGI, China). Three independent amplification experiments were performed for each sample. We sequenced 3 to 5 clones from each independent set of amplification and cloning, so there were a total of 9 to 15 clones for each sample. Bisulfite sequencing data and the C–T conversion rate were analyzed by BIQ Analyzer software [69]. To ensure high data quality, only sequences that had a C–T conversion rate >95% were included. Methylation data from bisulfite sequencing were evaluated by computing the percentage of methylated CpGs of the total number of CpGs.

### Experimental design

#### Experiment 1

SCNT embryos were treated with 0, 0.05, 0.5, 1 or 5 µM Oxamflatin post-ionomycin for 12 h. MII oocytes from the same batch without treatment were *in vitro* fertilized and used as an additional control group. *In vitro* development to 2-cell, 4-cell, morula, and blastocyst stages was monitored at 48, 72, 120, and 168 h of culture, respectively (0 h being the time embryos were transferred to G1.3).

SCNT embryos were treated with 1 µM Oxamflatin post-ionomycin for 0, 6, 12, 18, and 24 h to optimize the treatment of Oxamflatin. The developmental rates of blastocyst were tested at 168 h of culture.

#### Experiment 2

IVF embryos (IVF group) and SCNT embryos treated with 0 µM (C-NT group) or 1 µM Oxamflatin (T-NT group) were collected at the 2-cell, 4-cell, 8-cell, and blastocyst stage for detecting the acetylation level on H3K9 and H3K18.

#### Experiment 3

The total, TE, and ICM cell numbers in blastocysts of the three groups were estimated to assess the quality of Oxamflatin-treated SCNT blastocysts. The cell numbers in blastocysts were estimated by counting the total number of nuclei using DAPI. The number of trophectoderm (TE) nuclei was estimated using immunostaining for CDX2. The cell number of the ICM was assessed as the total number of nuclei minus the number of TE nuclei [28].

#### Experiment 4

The rate of cell death in day 7 blastocysts was examined by TUNEL assay to assess the quality of Oxamflatin-treated SCNT blastocysts.

#### Experiment 5

The relative expression levels of apoptosis-related genes (*Bax*, *Bax inhibitor*, *Bcl-XL*, *Survivin*, and *Caspase-3*) and development-related genes (*OCT4*, *NANOG*, *SOX2*, and *CDX2*) in blastocysts were compared among the three groups.

#### Experiment 6

The DNA methylation status of *satellite I* was analyzed in blastocysts of the three groups using BSP.

### Statistical analysis

Outcomes were tested by one-way ANOVA and LSD tests using the SPSS 13.0 software (SPSS Inc., Chicago, IL, USA). Differences were considered significant at P<0.05. Data were presented as mean ± SEM.
